# Preliminary Results on the Evaluation of the Occurrence of Tetrodotoxin Associated to Marine *Vibrio* spp. in Bivalves from the Galician Rias (Northwest of Spain)

**DOI:** 10.3390/md16030081

**Published:** 2018-03-06

**Authors:** Jose Manuel Leão, Antonio Lozano-Leon, Jorge Giráldez, Óscar Vilariño, Ana Gago-Martínez

**Affiliations:** 1 Department Analytical and Food Chemistry, University of Vigo, 36310 Vigo, Spain; leao@uvigo.es (J.M.L.); amcbeluso21@gmail.com (A.L.-L.); jgiraldez@uvigo.es (J.G.); ovilarino@uvigo.es (Ó.V.); 2 EU Reference Laboratory for Marine Biotoxins, 36310 Vigo, Spain; 3 Laboratory ASMECRUZ, 36939 Bueu, Pontevedra, Spain

**Keywords:** tetrodotoxin, *Vibrio*, NRPS, PKS, HILIC-LC-MS/MS, bivalve molluscs

## Abstract

Tetrodotoxins (TTX) are a potent group of natural neurotoxins putatively produced by symbiotic microorganisms and affecting the aquatic environment. These neurotoxins have been recently found in some species of bivalves and gastropods along the European Coasts (Greece, UK, and The Netherlands) linked to the presence of high concentrations of *Vibrio*, in particular *Vibrio parahaemolyticus*. This study is focused on the evaluation of the presence of *Vibrio* species and TTX in bivalves (mussels, oysters, cockles, clams, scallops, and razor clams) from Galician Rias (northwest of Spain). The detection and isolation of the major *Vibrio* spp. and other enterobacterial populations have been carried out with the aim of screening for the presence of the pathways genes, poliketide synthase (PKS) and non-ribosomal peptide synthetase (NRPS) possibly involved in the biosynthesis of these toxins. Samples containing *Vibrio* spp. were analyzed by biochemical (API20E-galery) and genetic tests (PCR-RT). These samples were then screened for TTX toxicity by a neuroblastoma cell-based assay (N2a) and the presence of TTX was further confirmed by LC-MS/MS. TTX was detected in two infaunal samples. This is the first confirmation of the presence of TTX in bivalve molluscs from the Galician Rias.

## 1. Introduction

Tetrodotoxin (TTX) is a potent neurotoxin responsible for the highest fatality rate of all marine intoxications, commonly associated with different species of pufferfish. In particular, it is found mainly in the organs of fish from the Tetraodontidae family, as well as other marine species, such as the blue-ringed octopus (*Hapalochlaena* sp.) and gastropods [1,2]. Tetrodotoxin blocks sodium conductance by binding extracellularly at receptor-site one of sodium channels to occlude the outer pore and thereby prevent access of monovalent cations to the pore [1,3,4,5,6]. Clinical effects include a range of neuromuscular symptoms (such as paresthesia of the lips and tongue, dizziness, headache, and gastrointestinal symptoms). More severe symptoms include ataxia, incoordination, cardiac arrhythmias, seizures, and respiratory failure, leading to death [7].

Recent emergence of tetrodotoxin in bivalve molluscs in different locations across Europe, such as the United Kingdom [8], Greece [9], and The Netherlands [10], has generated the necessity of exploring other areas in Europe in order to investigate the presence of TTX. Moreover, the European Food Safety Authority (EFSA) recently published an opinion on TTX [7], in which a recommendation regarding the need of more occurrence data on TTX and its analogues in edible parts of marine bivalves from different EU waters to provide a more reliable exposure assessment was included. Furthermore, the panel states that a concentration lower than 44 µg TTX equivalents/kg shellfish meat is not expected to lead to adverse effects in humans.

Hypotheses on the occurrence of TTX include the TTX biosynthesis by host-associated bacteria TTX and a range of TTX analogues are evidenced to be produced by a wide range of bacterial species, including those from the genera of *Vibrio*, *Bacillus*, *Aeromonas*, *Shewanella*, *Alteromonas*, and *Pseudomonas* as reviewed in [11]. The link of TTX with marine phytoplankton was suggested by [9], who noted a correlation between TTX occurrence in shellfish and the prevalence of *Prorocentrum minutum* in seawater. Furthermore, TTX-like compounds were found in *P. minutum* cultures [12].

Structurally, TTX consists of a guanidium moiety connected to a highly-oxygenated carbon backbone.

The biosynthetic pathway of TTX remains elusive, but the hypotheses suggested by [13] presume the incorporation of a guanidinium moiety in TTX through an amidinotransferase (AMT) or non-ribosomal peptidesynthetase (NRPS) module, incorporating arginine, could be taken into account. Another possible explanation for the biosynthetic mechanism of TTX is the hypothesis proposed by [14] of TTX being assembled by a hybrid polyketide synthase (PKS)/non-ribosomal peptide synthetase (NRPS) enzyme complex that possibly incorporates an amidinotransferase (AMT). Nevertheless, none of the hypotheses above mentioned have been experimentally supported.

The association of the occurrence of TTX in bivalve molluscs to high concentrations of *Vibrio parahaemolyticus* has been recently reported [8] and the hypothesis for our study was, therefore, based on that observation.

*Vibrio parahaemolyticus* is a typical warm-water pathogen and, globally, is the leading bacteriological cause of illness associated with seafood consumption [15]. Infections are generally rare across Europe with a single exception, the northwest of Spain (Galicia), where cases and outbreaks have been reported on a regular basis since the late 1990s [16,17,18,19]. The pathways’ genes, PKS and NRPS, were indicated as genes involved in the biosynthesis of toxins [2,13] and for this reason their determination was also included in the objectives of this work, after isolation of the major *Vibrio* spp. and other enterobacterial populations.

Moreover, in order to accomplish the EFSA recommendation regarding the evaluation of the occurrence of TTX in bivalves from EU coastal areas, the main objective of this work was to investigate the possible presence of TTX in different bivalve molluscs from the Galician Rias, in the Atlantic west coast of Spain, in which the production of bivalves, and, in particular, mussels, is the most representative in the EU, also being one of the main areas of mussel production in the world.

**Figure 1:**
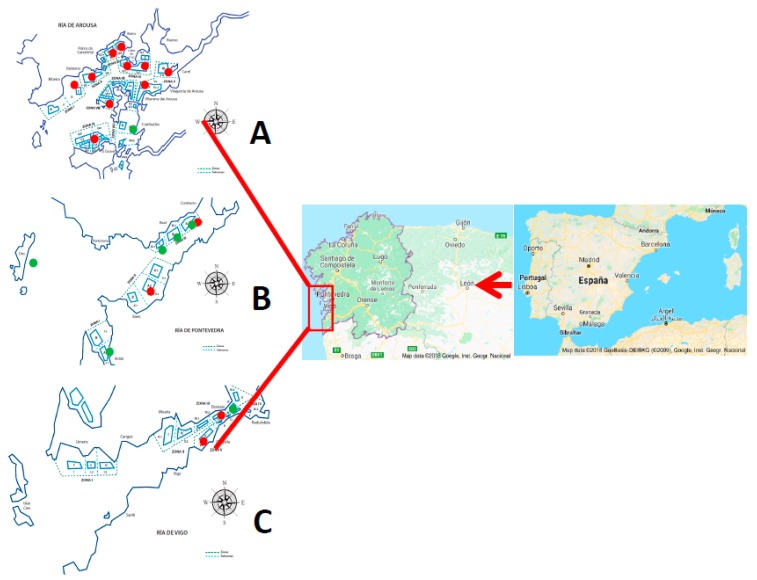
Geographical areas selected for the study and sampling (red: pelagic sites; green: infaunal sites) stations in (**A**) Ria of Arosa; (**B**) Ría of Pontevedra; and (**C**) Ría of Vigo.

Three main Rias of Galicia (Vigo, Pontevedra, and Arosa) were selected for this study (Figure 1).

The Galician Rias are estuary inlets similar to small fiords which extend from east to west and were formed by the sinking of river beds. Therefore, each Ria joins at least one river at its inland point, which is usually the main source of freshwater. The selected Rias extend over more than 300 km of coastline and cover an area of 670 km^2^. Mollusc cultivation is extensive in the estuarine portions of these Rias, with mussel production exceeding 200,000 tons per year. Mussels are grown on 15-m-long ropes hung from floating platforms and the infaunal bivalves (oysters, scallops, razor clams, cockles, and clams) are harvested in the estuarine zones around the Rias.

The harvesting areas selected for the study were those classified as A or B, taking into account in Regulation (EC) No. 854/2004. Additionally, the period of sampling started in January 2017 and ended on September 2017, and different types of bivalves (mussels, oysters, cockles, clams, scallops, and razor clams) were also included in the sampling plan.

The collected samples were first submitted to the laboratory for microbiological analysis, which consisted of the detection and isolation of *Vibrio* spp., and further confirmation of the presence of biosynthesis genes by PCR.

Samples with the presence of *Vibrio* spp. were further screened for TTX toxicity by a neuroblastoma cell-based assay (N2a) [20,21] and the TTX was confirmed by hydrophilic interaction liquid chromatography coupled to tandem mass spectrometry (HILIC-LC-MS/MS) under the conditions proposed by [20,22,23] and further modified by [24].

## 2. Results

### 2.1. Microbiological Results

The presence of *Vibrio parahaemolyticus* was detected in all sampling points and throughout the entire period of study. *Vibrio* spp. was detected in 286 out of 1279 bivalves investigated. A higher presence was observed in summer and autumn seasons (at a range of temperatures from 19.9 to 20.4 °C). Data on the distribution of *Vibrio* spp. are shown in Table 1.

A total of 515 strains (89—*V. parahaemolyticus*; 403—*V. alginolyticus*, 2—*V. fluvialis*, 5—*V. cholerae*, 1—*V. vulnificus*, 1—*Photobacterium damselae*, 4—*Aeromonas* spp., and 10—*Shewanella* spp.) were identified in the samples evaluated in this study. *Vibrio* strains were submitted to investigation of genes associated with the biosynthesis of toxins (PKS-NRPS). No fragment amplification was observed for PKS genes in the strains evaluated, while the NRPS gene was found in 53 strains. Similar results regarding the association of the NRPS gene with the biosynthesis of toxins was previously found by [14].

### 2.2. Cell Assay (N2a) Results

**Figure 2:**
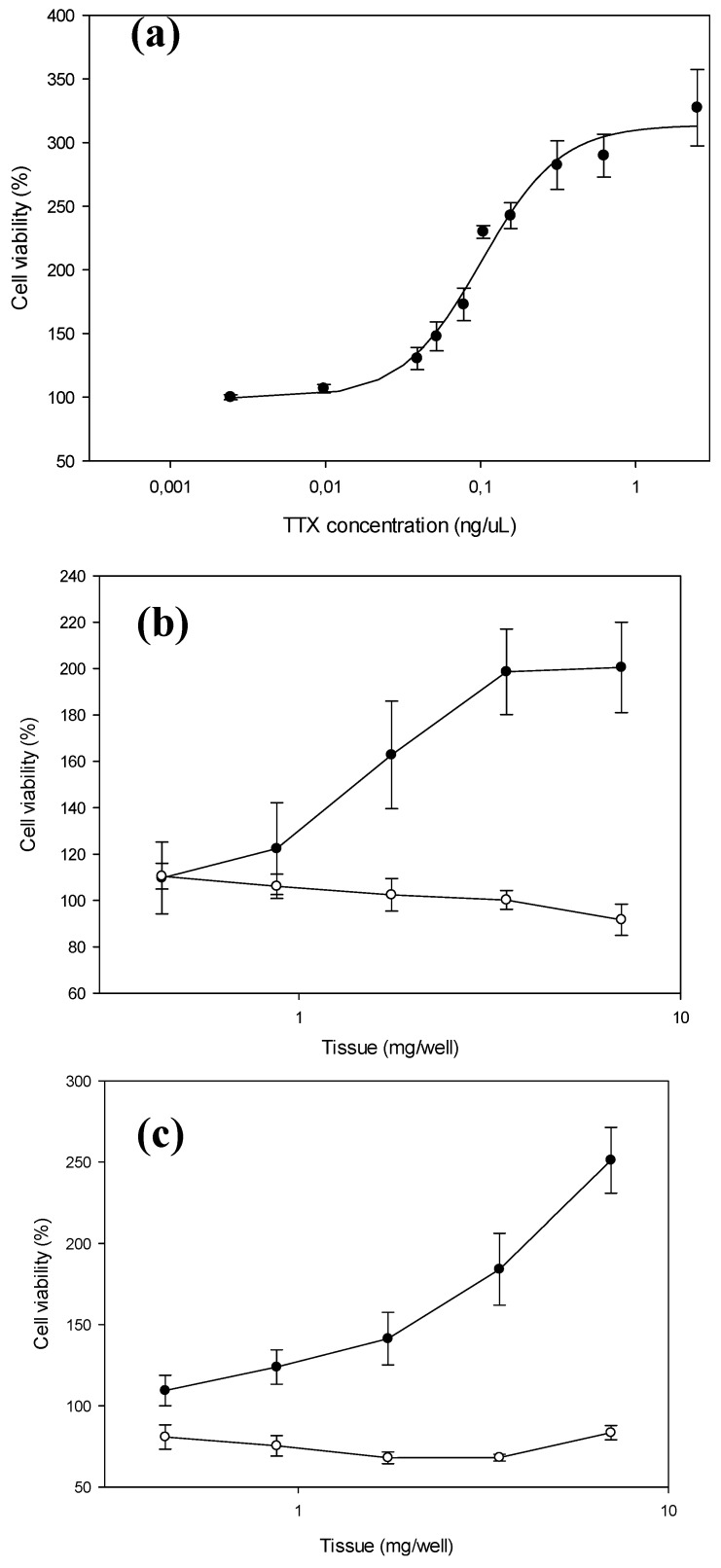
Dose response curves of standard of tetrodotoxin (**a**) and samples; oyster (**b**); and cockle (**c**). Wells with ouabain/veratridine (O/V) treatment are in black and wells without O/V treatment are in white.

A total of 286 bivalve samples with *Vibrio* spp. were screened for TTX toxicity by N2a assay. The presence of TTX toxicity was found in 2 out of 286 samples by N2a (cockles from Ria of Pontevedra collected in June 2017 and oysters from Ria of Arosa collected in February 2017) (Figure 2).

### 2.3. HILIC-MS/MS Results

All sample extracts previously analysed by N2a were analysed by HILIC-MS/MS under the optimized conditions described in Section 4.4, the qualitative and quantitative *m*/*z* transitions used in this study being 162.1 and 320.1. Additional *m*/*z* transitions mentioned in the experimental section were also monitored to investigate the potential presence of TTX analogues.

**Figure 3:**
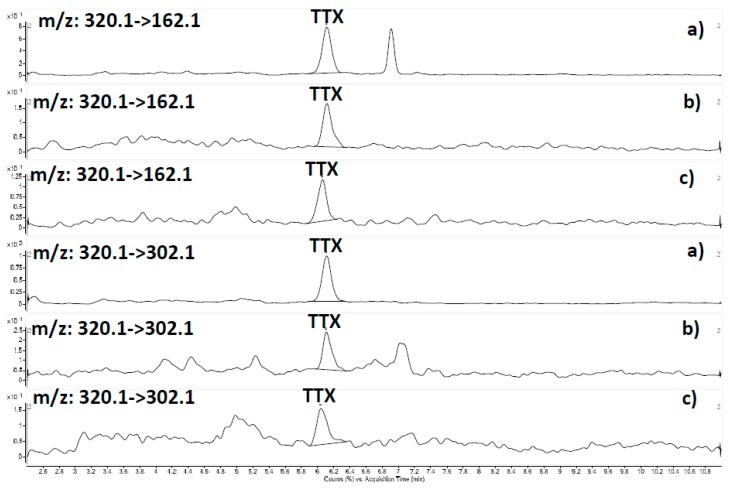
HILIC-MS/MS results: (**a**) matrix match standard of 0.2 µg of TTX/Kg; (**b**) cockle extract; and (**c**) oyster extract.

Good agreement was observed with the results obtained by N2a, since no presence of TTX or any of the TTX analogues were detected in most samples analysed, except for two samples from infaunal areas (cockle and oyster) in which the presence of TTX was confirmed by using the *m*/*z* transitions mentioned above (Figure 3b,c). The confirmation of TTX was carried out by using standard addition of TTX to uncontaminated mussel tissue, comparing retention times and primary and secondary MRM peaks. The absence of a second peak in the qualifying transition (320.1→162.1) allows one to conclude that the second peak obtained for the matrix match standard (a) corresponds to an interference (I) from the matrix (Figure 3a). No TTX analogues were found in these samples. The quantification of TTX was performed against the TTX certified reference standard using a matrix match calibration in the range 0.20–10 µg/Kg. The concentration of TTX in samples was 2.3 µg/Kg in cockles (Figure 3b) and below the LOQ (LOQ = 0.9 µg/Kg) in oyster sample (Figure 3c) No presence of TTX or any of its analogues was found in any of the mussel samples selected for this study.

## 3. Discussion

Several species of *Vibrio* spp. (*V. alginolyticus*, *parahaemolyticus*, *cholerae*, *fluvialis*, and *vulnificus*) were found in the samples selected from the different harvesting areas included in this study, with *V. alginolyticus* being the predominant species in most areas throughout the period of evaluation. These results are in agreement with those previously reported in [18].

*Vibrio* spp. were found in 286 bivalve samples out of 1279 from the selected areas during the period of January–September 2017. The seasonal distribution is also in accordance with previous studies carried out in the same geographical area [17,18,19] in which a higher density of *Vibrio* spp. were found in seasonal periods with warmer water temperatures. Among the *Vibrio* spp. isolated, a total of 53 strain contained the NRPS gene, which, according to other authors, is associated with the biosynthesis of toxins in bacteria [11]. The presence of the NRPS gene has been also observed in the samples where TTX was detected; nevertheless, this presence was also observed in other *Vibrios* isolated from samples where TTX was not detected. Therefore, the association of this gene to the production of TTX is not clear and needs further investigation. In any case, this is the first report on the identification of the NRPS gene in bacteria isolated in bivalves from the Galician Rias.

Those bivalve samples where the presence of *Vibrio* spp. were detected were analysed by N2a and only two samples from infaunal areas (cockle and oyster) showed TTX toxicity response. A clear relationship for the dose-response between absorbance (living cells) and concentration of the toxin was obtained after incubation for 20 h. The cell assay showed great sensitivity, with a positive response at very low toxicity levels, resulting in a very good alternative for a sensitive screening of TTX toxicity-like compounds in bivalve samples, although modifications, in particular those related to the reduction of the analysis time and matrix effects, are still needed.

The results obtained after the application of HILIC-LC-MS/MS under the optimized conditions described in this work confirm the presence of TTX in the two samples found positive by N2a, in which the presence of *Vibrio* spp. codified for the NRPS gene had been also found. No TTX analogues were found in these samples, nor in any other sample selected for the study. Two transitions were used for the quantitation and confirmation of TTX analogues. The quantitative transition specific for TTX corresponds to the fragment with *m*/*z* 320.1→302.1, the product ion consists of a water loss from the protonated molecular ion, and the qualitative ion pair with *m*/*z* 162.1 (2-aminohydroquinazoline) corresponds to the fragment ion commonly used for most TTX analogues. The transition [M + H]^+^→[2-aminohydroquinazoline] was used to screen for TTX and its analogues.

The TTX concentration levels found in the positive samples were low according to the recommendation limit established by EFSA (44 µg/Kg) and, as it was previously mentioned, TTX was only found in infaunal samples. These samples had been collected in February and June from intertidal areas, with water temperatures of 13.5 °C and 17 °C, respectively, and salinity levels around 35‰. The areas where these samples were collected, as well as the environmental conditions, are similar to those described by [25] for the majority of TTX-positive shellfish found in the UK. No TTXs were found in the pelagic samples evaluated in this study.

## 4. Materials and Methods

### 4.1. Sampling Plan

A sampling plan was designed, including pelagic and infaunal sites from the selected harvesting areas described in the introduction. These areas are also described in Figure 1. From January to September 2017, a total of 1.279 samples of molluscs were collected weekly; 304 samples collected in Ría de Vigo, 109 in Ría Pontevedra, and 866 in Ría Arosa.

### 4.2. Microbiological Analysis

The collected samples were placed in sterile bags and transported in refrigerated trucks (12 °C) to the laboratory to be processed for analysis in less than one hour. Samples were removed from the bags and washed in running potable water. Dead shellfish, or those with broken shells, were discarded.

For the microbiological analysis of *Vibrio*, 25 g of bivalve samples obtained from 12 to 15 individuals (meat and liquor) were weighted, and 225 mL of alkaline saline peptone water (ASPW, OXOID, Hampshire, UK) were added. The matrix was homogenized for 2 min in a Stomacher (IUL, Barcelona, Spain), and directly incubated at 37 °C for 18 h. After incubation, a 3 mm loopful from the top 1 cm of each broth showing growth was streaked on different plates: thiosulfate-citrate-bile salt-sucrose (TCBS) agar (Oxoid, Hampshire, UK), and ChromAgar Vibrio (ChromAgar, Paris, France). The plates were incubated at 37 °C for 24 h. At least five typical sucrose-negative and positive colonies from each plate (TCBS), and mauve and white colonies from ChromAgar Vibrio were isolated and subjected to identification by biochemical tests on API 20E strips (bioMérieux, Marcy-l’Etoile, France) and other complementary biochemical assays. The isolated strains were stored at −80 °C for further analysis.

### 4.3. Investigation of PKS-NRPS Gene Fragments

Isolates were screened for the presence of biosynthesis genes. The presence of NRPS was determined by PCR using two sets of degenerate primer A2gamF/A3gamR designed from conserved regions of adenylation domains and MTR/MTF to amplify the conserved A domain of the non-ribosomal peptide synthetase gene cluster, according to [26]. Other degenerate primers were used for PKS, according to [27].

For DNA extraction, the isolate was cultured overnight on a tryptone soy agar plate containing 3% NaCl at 37 °C. Several well-grown colonies were chosen and resuspended in 300 μL sterile distilled water and boiled for 15 min to lyse the cells. The lysate was centrifuged, and the supernatant containing DNA was used directly as a template in the PCR. PCR was carried out in a PTC200 thermocycler (MJ Research, Waltham, MA, USA) with the following reaction conditions: denaturation at 94 °C for 3 min, followed by 35 cycles of denaturation at 94 °C for 1 min, primer annealing at 60 °C (A2gamF/A3gamR); 55 °C for MTR/MTF for 1 min, and primer extension at 72 °C for 1 min. A final extension was performed at 75 °C for 7 min. The amplicons were analysed in a 1.8% agarose gel.

The gels were visualized under UV light. The set A2gamF/A3gamR amplifies fragments of 300 bp. The set MTR/MTF amplifies fragments between 750 and 1000 bp. The PCR recorded positive when a band of the appropriate size was visualized.

### 4.4. N2a Assay

N2a cell assay is based on the toxins blocking the cellular swelling and death resulting from the veratridine enhancement of sodium influx into the mouse neuroblastoma cell line in the presence of ouabain. The assay enabled the semi-quantitation of TTX based on the percentage of living cells remaining.

The cell assay was performed using neuro-2a cells purchased from the American Type Culture Collection (ATCC; CCL-131) and cultured in 75 cm^2^ culture flasks containing 25 mL RPMI-1640 medium (R8758, Sigma-Aldrich, Irvine, UK) supplemented with 10% foetal bovine serum (F2442, Sigma-Aldrich, St. Louis, MO, USA), 1 mM sodium pyruvate solution (S8636, Sigma-Aldrich, Irvine, UK), 2 mM L-glutamine solution (G7513, Sigma-Aldrich, Irvine, UK), and 1000 units per litre of penicillin-streptomycin (P4558, Sigma-Aldrich, St. Louis, MO, USA). The cell line was routinely maintained in a humidified incubator at 37 °C under 5% CO_2_ (Forma Scientific, Inc., Model 3111, Marjeta, OH, USA). The conditions used in this assay were proposed by Manger et al. [20,21] with slight modifications to accommodate the assay to the detection of TTX. A standard of tetrodotoxin (TTX) was purchased from Tocris-Bioscience (Bristol, UK), Batch 43B, MW: 319.27 g/mol (C_11_H_17_N_3_O_8_). The same conditions as described by Turner et al. [28], with slight modifications, were used for the extraction of TTX for both N2a and LC-MS/MS, and the last step of dilution with acetonitrile required for the LC-MS/MS analysis, was not carried out for N2a, to avoid possible interferences in the cell assay. Cell cultures were incubated for approximately 24 h in 96-well culture plates. Culture wells received sample dilutions, which were tested in replicates of four wells. Ouabain (O3125, Sigma-Aldrich, St. Louis, MO, USA) and veratridine (V5754, Sigma-Aldrich, St. Louis, MO, USA) were also added and cells were incubated for 20 h. Cell viability was measured by the colorimetric method using tetrazolium (MTT, M5655, Sigma-Aldrich, St. Louis, MO, USA) metabolism. Plates were read on a spectrophotometer (Multiskan™ FC Microplate Photometer, Thermo Fisher Scientific Oy, Ratastie, Finland) at 570 nm for testing and 630 nm for reference.

### 4.5. LC-MS/MS Analysis

#### 4.5.1. Extraction

TTX extraction was carried out following the conditions described in [28] with slight modifications as mentioned in the previous section. Briefly, 5 g of shellfish homogenate were weighed into a 50 mL polypropylene centrifuge tube. 5 mL of 0.1% acetic acid (LC-MS grade, Fluka Analytical, Steinheim, Germany) was added to the sample and homogenized by vortex for 90 s. the centrifuge tube was capped and placed in a boiling water bath for 5 min, then cooled to room temperature and vortex-mixed for 90 s. The sample was centrifuged at 4000× *g* for 10 min., 1 mL of supernatant was transferred to a 1.5 mL centrifuge tube, and 5 µL of 25% ammonium hydroxide (LC-MS grade, Fluka Analytical, Steinheim, Germany) was added, vortexed, and centrifuged at 10,000× *g* for 60s for further clean up through graphitized carbon SPE cartridges (Supelclean ENVI CARB SPE tubes), 3 mL (0.25 g) from SUPELCO (Bellfonte, PA, USA) before HILIC-MS/MS analysis.

#### 4.5.2. SPE-ENVI-Carb Clean-Up

The conditions used for the clean-up were described by Boundy et al. [29] with slight modifications. Briefly, SPE-ENVI-Carb cartridges were conditioned with 3 mL of acetonitrile (LC-MS grade, Merck, Darmstadt, Germany):water (LC-MS grade, J.T. Baker, Center Valley, PA, USA) (80:20) containing 1% of acetic acid, followed by 3 mL of 0.025% of ammonium hydroxide. Four-hundred microliters of the sample extract were loaded into the conditioned cartridge and the eluent was discarded. Seven-hundred microliters of water were used to wash the stationary phase and the eluent was also discarded. Finally, 2 mL of acetonitrile:water (80:20) containing 1% of acetic acid were used for elution and 100 µL of eluate were diluted with 300 µL of acetonitrile and filtered with a 0.22 µm membrane filter (Millex, Millipore, Billerica, MA, USA) to be further analysed by HILIC-MS/MS.

#### 4.5.3. HILIC-LC-MS/MS Analysis

An Agilent 1290 Infinity LC system (Waldbronn, Germany) was used for the liquid chromatographic separation, which was performed on a HILIC column (ACQUITY UPLC Glycan BEH amide column 130A, 1.7 µm, 2.1 mm × 150 mm) from Waters (Dublin, Ireland), operating at 60 °C. The LC sample compartment was kept at 4 °C. Two microliters was the injection volume typically used for the LC-MS/MS analysis. The mobile phase for channel A consisted of water containing 75 µL of formic acid (LC-MS grade, Fluka Analytical, Steinheim, Germany) and 300 µL of 25% ammonium hydroxide and, for channel B, of acetonitrile:water (7:3) that contains 100 µL of formic acid. Table 2 summarizes the chromatographic conditions used in the analysis of TTX.

A 6495 Triple Quad MS/MS (QQQ) equipped with an iFunnel Jet Stream ESI (ESI) source (Waldbronn, Germany) was used for the analysis of TTX, being selected as the major fragment ion at *m*/*z* 162 to detect TTX and its analogues in MRM (multiple reaction monitoring) mode by detecting *m*/*z* transitions in tandem mass spectrometry, which are included in Table 3 [24].

Mass spectrometric conditions were optimised under the positive mode using TTX and 4,9-anhydro-TTX standard solutions with a concentration 81.2 µmol/Kg and 9.92 µmol/Kg, respectively, from CIFGA Laboratorio S.A. (Lugo, Spain), Batch: 12-001 and tetrodotoxin (TTX) standard solution from Tocris-Bioscience (Bristol, UK), Batch 43B, MW: 319,27 g/mol (C_11_H_17_N_3_O_8_). The optimised conditions are summarised in Table 4.

## 5. Conclusions

This is the first report on the occurrence of TTX in bivalves from the Galician Rias to the northwest of Spain. The concentration levels of TTX in the positive samples were significantly lower than those recommended by EFSA. The association of the presence of TTX in bivalve samples, with the presence of *Vibrio* spp. carriers of NRPS, as reported by other authors, has been also found in the samples where TTX was detected, nevertheless the presence of the NRPS gene was also observed in other *Vibrios* isolated from samples where TTX was not found. Therefore, the association of this gene to the production of TTX is not clear and needs further investigation. The data on TTX occurrence obtained in this study do not allow the conclusion that TTX represents a risk for public health in Galicia, at least for the time being; nevertheless, additional data are still required and more studies are in course to obtain more occurrence data. The evaluation of specific environmental conditions that could be associated with the biosynthesis of TTX and its analogues is also a future perspective on the research project that is being carried out.

## Figures and Tables

**Table 1:** Distribution of *Vibrio* spp. in the main Rias of Galicia.

Rias	Samples			
	Number Tested	With *Vibrio* spp. (%)	Pelagic *	Infaunal **
*Arosa*	866	20	171	2
*Pontevedra*	109	37	17	23
*Vigo*	304	24	70	3

* Mussel, ** Oyster, clam, razor clam, cockle, scallop.

**Table 2:** HILIC chromatographic conditions for TTX analysis.

Time (min)	A (%)	B (%)	Flow Rate (mL/min)
0.0	2.0	98.0	0.4
5.0	2.0	98.0	0.4
7.5	50.0	50.0	0.4
9.0	50.0	50.0	0.5
9.5	5.0	95.0	0.5
9.8	2.0	98.0	0.8
10.6	2.0	98.0	0.8

Mass spectrometry conditions.

**Table 3:** MS/MS conditions (MRM mode) for the analysis of TTX and TTX analogues.

Compound	Precursor Ion	Product Ion	Collision Energy (V)
TTX/4-epi-TTX	320.1	302.1	28
TTX/4-epi-TTX	320.1	162.1	44
11-deoxy-TTX/5-deoxy-TTX	304.1	286.1	28
11-deoxy-TTX/5-deoxy-TTX	304.1	162.1	44
4,9-Anhydro TTX	302.1	284.1	28
4,9-Anhydro TTX	302.1	162.1	44
6,11-dideoxy-TTX	290.1	272.1	28
6,11-dideoxy-TTX	290.1	162.1	44
5,6,11-trideoxy-TTX	272.1	254.1	28
5,6,11-trideoxy-TTX	272.1	162.1	44

**Table 4:** iFunnel and Jetstream ion source conditions for TTX analysis.

Source Parameters			
Gas temp (°C)	150	Polarity	Positive
Gas flow (L/min)	12	Fragmentor (V)	380
Nebulizer (psi)	45	Cell accelerator (V)	5
Sheath gas heater (°C)	400	Dwell (ms)	20
Sheath gas flow (L/min)	12	Delta EMV (V)	400
Capillary (V)	4000		
V charging (V)	300		
Ion Funnel Parameters (V)			
Pos high pressure RF	150	Neg high pressure RF	90
Pos low pressure RF	60	Neg low pressure RF	60
